# Epidemiological Profile of Otomycosis at the Peace Hospital of Ziguinchor (Senegal)

**DOI:** 10.3390/jof11030218

**Published:** 2025-03-12

**Authors:** Abdoulaye Diop, Hussein Younes, Papa Samba Diop, Kalilou Diallo, Youssouph Sambou, Khadim Diongue, Mouhamadou Ndiaye, Mame Ngoné Coly, Habibou Sarr, Evelyne Siga Diom, Daouda Ndiaye

**Affiliations:** 1 Health Science Training and Research Unit, Assane SECK University of Ziguinchor, Ziguinchor 27000, Senegal; kalildiall@yahoo.fr (K.D.); m.coly@univ-zig.sn (M.N.C.); h.sarr@univ-zig.sn (H.S.); es.diom@univ-zig.sn (E.S.D.); 2 PEACE Hospital of Ziguinchor, Ziguinchor 27000, Senegal; toyounes1989@hotmail.com (H.Y.); sambadiop91@yahoo.fr (P.S.D.); 3 Ziguinchor Regional Hospital Center, Ziguinchor 27000, Senegal; youssambou@gmail.com; 4 Service of Parasitology-Mycology, Faculty of Medicine, Pharmacy and Odontology, Cheikh Anta Diop University, Dakar 16477, Senegal; khadimase@gmail.com (K.D.); mouhamadou.ndiaye@ucad.edu.sn (M.N.); dndiaye23@gmail.com (D.N.)

**Keywords:** otomycosis, Senegal, *Candida*, *Aspergillus*, risk factor

## Abstract

The investigation of the fungal etiologies of otomycoses is a rare occurrence in Senegal. The present study aspires to ascertain the profile of these mycoses within the confines of Ziguinchor. Conducted from 3 February 2021 to 31 August 2022, this retrospective descriptive study encompassed a total of 82 patients presenting with clinically suspected otomycosis within the otolaryngology (ENT) department of the Ziguinchor Peace Hospital (ZPH). In this study, two samples were collected from the external auditory canal (EAC) of each patient using sterile swabs. These samples were first observed by direct microscopy and then cultured at 30 °C on Sabouraud chloramphenicol with or without cycloheximide. The identification of the isolates was based on their macroscopic, microscopic, and physiological characteristics. The mycological examination was positive in 70 patients, with a prevalence of 85.37%. The most prevalent fungal isolates were *Aspergillus section Nigri* (30%), *Aspergillus section Flavi* (20%), and *Candida albicans/Candida dubliniensis* (10%). Of the clinical signs examined, auricular pruritus (*p* = 1.7033 × 10^−6^) was the only one to demonstrate a positive correlation with the onset of otomycosis. These results indicate that fungal agents play a significant role in the pathogenesis of otitis externa, underscoring the importance of mycological diagnosis in ensuring optimal patient management.

## 1. Introduction

Otomycoses are ear infections caused by microscopic fungi, which develop mainly in the external auditory canal (EAC). According to recent studies, otomycosis represents 5 to 10% of all cases of otitis externa [1,2,3]. The most likely factors causing otomycosis are heat, humidity, poor personal hygiene, bathing or diving in cold water or seawater, and topical antibiotics [2,4,5,6].

The incidence of these infections has increased in recent years due to the widespread use of antibiotics to treat bacterial ear infections, as well as other factors linked to weakened immunity [2]. The most commonly encountered agents are species belonging to the *Aspergillus* and *Candida* genera [2,7].

The relative frequency of etiological agents can vary depending on geographical factors [2,8]. Otomycosis emerges frequently in tropical and subtropical zones, where it is unfortunately often confused with chronic otitis media because of its association with common germs. In Senegal, there are very few studies on otomycosis, and those existing are limited to the Dakar region. Consequently, prescribing physicians must address the challenge of selecting an appropriate therapeutic plan due to the diverse polymorphism of fungal agents implicated in otomycosis. In order to optimize the choice of antifungal medication and significantly reduce the risk of serious complications, it is first necessary to determine the pathogens responsible for otomycosis. The present work, therefore, aims to determine the prevalence of otomycosis in the region of Ziguinchor (Senegal), to identify the etiological agents and the factors likely to be associated with its occurrence.

## 2. Materials and Methods

This is a retrospective study with descriptive and analytical aims. The study encompassed all patient files received for ear sample testing from 3 February 2021, to 31 August 2022. The objective was to make a mycological diagnosis of otomycosis, with meticulous record-keeping. The studied data were obtained from a database recorded in the laboratory’s dedicated register, and from otomycosis sample reception forms. Missing data were filled-in from otomycosis sample forms and patient files held in the otorhinolaryngology (ENT) department of the Ziguinchor Peace Hospital.

During the study period, the mycological diagnosis of otomycoses was based on swab samples taken from the otorhinolaryngology (ENT) department of the Ziguinchor Peace Hospital followed by the mycological examination of the said samples by the laboratory of the Ziguinchor Regional Hospital Center (ZRHC).

Samples were collected by an ENT physician before any administration of antifungal agents in all patients with clinically suspected otomycosis. The EAC was first cleaned with a sterile cotton swab (Deltalab Barcelona Spain) soaked in 0.9% NaCl (Laboratoires Physiodose Gilbert Caen France). Ear samples were obtained either under a headlamp or an otomicroscope, using two sterile swabs soaked in 5 mL of 0.9% NaCl (Laboratoires physiodose Caen France). After collection, each sample was promptly placed in a plastic bag and securely sealed, then handed over to the patient, who transported it at room temperature within 20 min to the laboratory of the laboratory of the Ziguinchor Regional Hospital Center (ZRHC).

For each sample collected, a mycological examination was conducted immediately after reception in the following stages:

A direct microscopic examination of one of the two swabs was performed in a drop of saline solution. To do this, 500 µL of saline was added to the swab, which was vortexed. Then, 70 µL of the vortexed sample was used for direct slide-to-slide examination, which was observed with 10× and then 40× objectives.

The inoculation of the remaining swab onto Sabouraud Chloramphenicol (SC) HIMEDIA^®^ agar (HIMEDIA Laboratories, Mumbai, India) and Sabouraud Chloramphenicol Cycloheximide) HIMEDIA^®^ agar (HIMEDIA Laboratories, Mumbai, India).

The remaining swab was then inoculated onto Sabouraud Chloramphenicol (SC) HIMEDIA^®^ agar (HIMEDIA Laboratories, Mumbai, India) and Sabouraud Chloramphenicol Cycloheximide) HIMEDIA^®^ agar (HIMEDIA Laboratories, Mumbai, India). The inoculation process was adapted according to the nature of the sample. Samples with visible solid elements were collected with a loop, placed in a Petri dish, and cut with a scalpel blade. The cut-out pieces were then spot-applied to SC (HIMEDIA Laboratories, Mumbai, India) and SCA (HIMEDIA Laboratories, Mumbai, India) agars. For other samples, the swab was directly inoculated using the quadrant method on SC (HIMEDIA Laboratories, Mumbai, India) medium, followed by SCA (HIMEDIA Laboratories, Mumbai, India). The previously inoculated agar media (SC and SCA) were then incubated in a proofer at 30 °C with 5% CO_2_ in a humidified atmosphere and observed daily for one to two weeks.

Identification of the pathogen was based on macroscopic (colony characteristics, appearance, and color) and microscopic criteria for filamentous fungi (thallus appearance, hyphal color, shape, spore morphology, and conidiogenesis) [9]. Yeasts were also identified based on the following criteria:

On a macroscopic level, the distinguishing features of the two yeast types are as follows: Candida yeasts manifest as white, shiny or matte, smooth or wrinkled colonies with no visible pigmentation, while *Cryptococcus* yeasts are beige, smooth, runny, or shiny.

Microscopic: In the case of the *Candida* genus, we observe oval blastopores with unipolar budding, measuring between 2 and 10 µm in length. These blastopores may or may not be accompanied by mycelium or pseudo-mycelium. In contrast, the Cryptococcus genus features globular blastospores with multipolar budding, ranging from 2 to 12 µm in diameter. These blastospores may also be associated with a capsule and the absence of mycelial filaments.

In the context of susceptibility and/or resistance to cycloheximide, the presence of growth on SCA agar previously incubated at 30 °C was sufficient to indicate that a species was resistant to cycloheximide. For yeasts, this was oriented towards certain species, including *Candida albicans/Candida dubliniensis*.

The Blastèse test is a method used to identify the presence of *Candida albicans/Candida dubliniensis*. The test is performed by adding one drop of yeast suspension to 1 mL of fresh human serum and incubating it at 37 °C for three hours. The presence of germ tubes indicates the presence of either *Candida albicans or Candida dubliniensis*.

India ink test: the first step is to apply 25 µL of India ink to an object slide, then adding 75 µL of 0.9% NaCl (physiodose Laboratoire Caen France) and a small portion of the colony. The next step is covering it with a coverslip to be read under the microscope at 40× objective. The presence of a capsule indicates the presence of *Cryptococcus* and excludes *Candida.*

Urease test: A whole colony isolated on SC medium is placed in a tube containing Bio-Rad urea-indole broth (Laboratoire Bio-Rad, Paris, France). This is incubated at 37 °C for 4 h. A positive test result is indicated by the medium turning purplish-red, confirming the genus *Cryptococcus*, and invalidating the diagnosis of *Cryptococcus* sp. [10].

The data collected were entered into Excel 2013 and analyzed using R software (R Core Team, R Foundation for Statistical Computing, Vienna, Austria (2022)). Variables such as sociodemographic (age, sex, geographic origin, etc.), clinical (history and clinical signs), and mycological (direct examination, isolated species) variables were analyzed for each patient. Data were described in terms of mean or percentage, with quantitative or qualitative variables. The prevalence rates were calculated overall and also according to age, sex symptoms, and contributing factors. Pearson’s χ2 test at the 5% significance level and Fisher’s exact test, when the numbers are less than 5, were used to compare the proportions.

## 3. Results

### 3.1. Characteristics of the Study Population

During the study period, 82 patients underwent mycological examination; their ages ranged from 7 months to 79 years, with a mean age of 37.81. The majority of patients were over 21 years of age, amounting to 78.04% of the study population, while 16 patients were under 20 years of age, representing 19.51% of the total (see Table 1). Females accounted for 53.66% of the study population, giving a sex ratio of 0.86.

### 3.2. Otomycosis: Prevalences and Frequencies Depending on the Variables Studied

Of the 82 samples taken, the mycology cultures were positive on 70 occasions, representing a prevalence rate of 85.37%, including three negative samples using direct examination. Two samples were positive using direct examination and turned out negative on culture. The culture allowed the isolation of 82 strains, including 67.07% molds (55 strains) and 32.93% yeasts (27 strains). The fungal agents most frequently encountered were *Aspergillus section Nigri* in 30% (21/70) of cases, *Aspergillus section Flavi* in 20% of cases, *Candida albicans/Candida dubliniensis* in 10% of cases, and *Candida* sp. in 10% of cases. In 12 cases (17.14%), the associations between two fungal species were noted, with the association between *Aspergillus section Nigri + Candida* sp. (7.14%) and that of *Aspergillus section Flavi* + *Candida* sp. (4.28%) (Table 2) being prominent.

Based on data from 70 positive cases, the prevalence of otomycosis was virtually identical in males and females (86.84% vs. 84.09%), with a *p*-value of 0.7252 (see Table 1). Thus, there was no significant difference in the incidence of otomycosis according to sex. Regarding age, the prevalence of otomycosis was 90.90%, 81.25%, and 80.65% in those aged 21–40, 0–20, and over 40 years, respectively, with a *p*-value of 0.9363 (see Table 1). Thus, there was no significant difference in the incidence of otomycosis according to age.

Clinically, the infection was unilateral for 80% of cases (right ear 47% and left ear 33%) and bilateral for 20% of cases. Regarding the symptoms associated with the infection, they were specified for 87% (61/70) of the patients. The predominant symptoms were ear pruritus (72.86%), otalgia (60%), hearing loss (40%), otorrhoea (21.43%), and tinnitus (20%). Other symptoms, such as fever (4.29%), autophony (1.43%), and headache (1.43%), were less frequent in patients with otomycosis (Table 2). Similarly, the main lifestyle-related factors found in otomycosis were the improper cleaning of ears (36 out of 70 patients or 51.43%), humidity (25.71%), wearing headphones (15.71%), swimming (8.57%), and the presence of foreign bodies in the EAC (7.14%). Other factors linked to patients’ medical history were also highlighted in 40 cases. These were mainly allergies (20%), taking local antibiotics (15.71%), diabetes (5.71%), and ear trauma (4.29%). Other medical factors such as HIV infection, prolonged corticosteroid therapy, otologic surgery, and traditional medicine were found with a frequency of 2.86% each (Table 3).

The analysis of the parameters does not highlight an association between sex (*p* = 0.7252), age (*p* = 0.9363), lifestyle (*p* = 0.382), medical history (*p* = 0.9645), certain clinical signs (ear pain, hypoacusia, tinnitus, fever, and headache…) and the occurrence of otomycosis. Furthermore, otomycosis was significantly correlated with auricular pruritus (*p*=1.7033 × 10^−6^).

## 4. Discussion

Mycological diagnosis is vital in curing otomycosis, as it helps guide the choice of treatment. A retrospective descriptive study was conducted at the Ziguinchor Peace Hospital, examining all patients with clinically suspected otomycosis during the study period. An ENT doctor took samples before the administration of antifungal agents. The survey revealed a prevalence of 85.37% for this disease, which is usually overlooked in everyday practice. The significance of mycological diagnosis in establishing a reliable diagnosis of this mycosis is now well established.

The high prevalence of 85.37% found in our study is comparable to that found by certain authors in Africa (80% in Côte d’Ivoire, 74.2% in Egypt) and in Asia (82% to 92% in Iran, 70% in Turkey, 78% in India) [7,11,12,13,14,15]. These data show that fungal infections occupy an important place in the etiologies of otitis externa. Conversely, lower prevalences have also been noted by other authors, such as 56.6% found in Senegal itself, 12.53% in Niger, 42.8% in Côte d’Ivoire, 50.5% in Iran in 2022, 19.4% in Brazil, and 28.4% in Spain [2,3,16,17,18]. The difference in prevalence observed in Senegal between our study and that of Dieng T et al. could be explained by several factors. Firstly, different climatic and weather conditions between Ziguinchor and Dakar could be a contributing factor. Secondly, most of our patients presented either medical history and/or lifestyle habits (promoting factors) that could favor the occurrence of otomycosis. The high prevalence observed in Senegal and other African countries, such as Côte d’Ivoire [7], can be explained by the combination of certain factors: the climate (humidity and heat), certain religious, cultural, or aesthetic practices that lead to maintaining a wet external auditory canal (ablutions, wearing a scarf or veil, and long locks braided with synthetic or real hair by women).

Fungal culture enabled the isolation of 82 strains of molds and yeasts, with a predominance of molds (67.07%). The dominant species were *Aspergillus section Nigri*, followed by *Aspergillus section Flavi*, and the *Candida albicans/Candida dubliniensis* complex. In addition to the aforementioned species, other types of the *Candida* genus were also frequently isolated; however, due to a paucity of resources, we were unable to identify them at the species level. However, such identification would provide a more accurate understanding of the fungi responsible for otomycosis in Ziguinchor. This would facilitate more effective infection management by determining the most efficacious antifungal agents. This represents a significant limitation of the present study.

This result is similar to that of Roohi B et al. in Iran who found that the dominant species was *Aspergillus section Nigri* (58.57%), followed by *Aspergillus section Flavi* (19.23%), and finally the species *Candida parapsilosis* (14.96%) [17]. Our result is different from that of Dieng T et al. in Senegal, which highlighted *Candida albicans* and *Aspergillus section Fumigati* as co-dominant species (26.30% each), followed by *Aspergillus section Nigri* (22.80%) and *Candida* sp. (12.30%) [2]. The distribution of species varies from one country to another, from one region to another. Thus, in Côte d’Ivoire, the main agents of otomycoses found by Bosson-Vanga H et al. are *Candida albicans*, *Aspergillus section Flavi* and *Candida parapsilosis* [16]. Just as in Poland, Kurnatowski et al. found a predominance of species of the genus *Candida*, particularly *Candida parapsilosis* (29.3%), *Candida albicans* (19.0%) and *Aspergillus section Nigri* (14.7%); however, a predominance of *candida* was reported by Pontes ZB, with *C. albicans* (30%), *C. parapsilosis* (20%) and *Aspergillus section Nigri* (20%). The results of Amrin K et al. in India highlighted a predominance of *Aspergillus*, with *Aspergillus section Nigri* at 43.73% and *Candida* sp. at 35.2%, and *Aspergillus section Fumigati* (1.06%) [18,19,20].

Regarding the microscopic examination, we found two positive samples on direct examination that were negative on culture. The reason for this may be that some of our patients were taking traditional medicines with known antifungal properties, which could have led to the elimination of the infection.

In our study and those of Adoubryn et al. [7] and Djohan et al. [21], neither sex nor age predicted otomycosis. In contrast to Ali et al. and Aboulmakarim et al., who found a predominance of females and a higher incidence in people aged between 21 and 40 years [22,23], Nwabuisi et al. found an equality between males and females [24] and Abdelazeem et al. found a predominance of males with a higher incidence in people aged between 21 and 40 years [25]. The incidence of otomycosis as a function of age and sex varies from study to study and is the source of much controversy.

In the present study, the most frequently cited factors related to otomycosis were determined to be improper ear cleaning (51.43%), humidity (25.71%), wearing headphones (15.71%), bathing (8.57%), and the presence of cotton in the CAE (7.14%). However, no significant association was identified between these factors and the occurrence of otomycosis. As emphasized in our study, the primary factor promoting otomycosis is the untimely cleaning of the ear with a cotton bud, as evidenced by the work of Djafarou AB et al. [3] in Niger (53.57%) and Abdoumakarim S et al. [23] in Morocco (56.4%). The utilization of poultry feathers for ear cleaning has been identified as a risk factor for otomycosis, attributable to the prevalence of avian aspergillosis in these animals. Conversely, a study by Garcia-Martos et al. in Spain demonstrated that swimming (90%) was the predominant risk factor for otomycosis [26]. Vennewald et al. also state that the risk of contracting otomycosis is five times higher in swimmers than in non-swimmers [27]. Medical history including allergies, otological surgery, and/or ear trauma was frequently found, although none of these were significantly associated with the occurrence of otomycosis. This result is consistent with several other studies [7,28,29,30].

According to the results of our study, the primary symptoms were primarily ear pruritus (72.86%), followed by otalgia (60.00%) and hypoacusis (40.00%). Statistically, auricular pruritus has been demonstrated to be associated with the onset of otomycosis. Similar results have been demonstrated by several authors, although differences in frequency have been noted [2,28,29].

Despite the inherent limitations of the study, including the unavailability of equipment and resources necessary for the more precise identification of the species and assessment of antifungal resistance, the study’s primary objective was successful. This objective was to determine the prevalence of otomycosis in the Ziguinchor region of Senegal, identify the underlying etiological agents, and determine the factors associated with its occurrence.

## 5. Conclusions

The survey revealed a relatively high prevalence of otomycosis cases in the southern region of Senegal. Given the predominance of the genera Aspergillus and Candida as etiological agents in otitis media, there is a need for mycological diagnosis in cases of otitis media to avoid the excessive use of antibiotics. It is important to carry out prevalence surveys on larger samples to better assess the impact of these fungal pathologies and the risks they represent.

## Figures and Tables

**Table 1:** Demographic data of patients with otomycoses.

Characteristics	Number of Patients Examined N	Number of Positive Patients n (%)	Prevalence en % n/N	*p*-Value
**Age range (years)**				0.9363
[0–20]	16	13 (18.57)	81.25	
[21–40]	33	30 (42.86)	90.90	
[41–79]	31	25 (35.71)	80.65	
Not specified	2	2 (2.86)	100.00	
**Total**	**82**	**70 (100)**	**85.37**	
**Gender**				
Male	38	33 (47.14)	86.84	0.7252
Female	44	37 (52.86)	84.09	
**Total**	**82**	**70 (100)**	**85.37**	

**Table 2:** Fungal species isolated from patients with otomycosis.

Species	Number	Percentage (%)
Mold		
*Aspergillus niger*	21	30.00%
*Aspergillus flavus*	14	20.00%
*Aspergillus* sp.	5	7.14%
*Aspergillus versicolor*	1	1.43%
*Rhizopus* sp.	1	1.43%
*Scopulariopsis* sp.	1	1.43%
Total	43	61.43%
Yeasts		
*Candida albicans/Candida dubliniensis*	7	10.00%
*Candida* sp.	7	10.00%
*Cryptococcus* sp.	1	1.43%
Total	15	21.43%
Co-infections		
*Aspergillus niger + Candida* sp.	5	7.14%
*Aspergillus flavus + Candida* sp.	3	4.28%
*Aspergillus niger + Candida albicans*	1	1.43%
*Aspergillus niger + Apergillus flavus*	1	1.43%
*Aspergillus niger + Cryptococcus* sp.	1	1.43%
*Aspergillus flavus + Cryptococcus* sp.	1	1.43%
Total	12	17.14%

**Table 3:** Clinical data of patients with otomycoses.

Characteristics	Frequency Among Patients Examined N	Frequency of Positive Patients n (%)	Prevalence n/N (%)	*p*-Value
Lifestyle				
Swimming	6	6 (8.57)	100.00	0.5826
Humidity	22	18 (25.71)	81.81	0.7227
In-ear earphones	12	11 (15.71)	91.60	0.6797
Improper cleaning	42	36 (51.43)	85.71	0.9271
Presence of cotton in the external auditory canal	5	5 (7.14)	100.00	1
History				
Allergies	15	14 (20.00)	93.3	0.4508
Local antibiotic treatment	12	11 (15.71)	91.6	0.6839
Ear trauma	5	3 (4.29)	60	0.1531
Diabetes	4	4 (5.71)	100	1
Prolonged corticosteroid therapy	2	2 (2.86)	100	1
HIV infection	2	2 (2.86)	100	1
Otologic surgery	2	2 (2.86)	100	1
Traditional medicine	2	2 (2.86)	100	1
No significant medical history	38	30 (42.86)		0.1265
Symptomatology				
Otalgia	49	42 (60.00)	85.71	1
Otorrhea	26	15 (21.43)	57.70	5.3389 × 10^−9^
Hypoacusis	32	28 (40.00)	87.50	0.7572
Ear pruritus	51	51 (72.86)	100	1.7033 × 10^−6^
Tinnitus	18	14 (20.00)	77.70	0.4490
Fever	4	3 (4.29)	75	0.4757
Autophony	1	1 (1.43)	100	1
Headache	1	1 (1.43)	100	1
Not specified	8	7 (10.00)	87.50	

## Data Availability

For reasons of confidentiality and ethics, these data are not accessible. However, if necessary, these data can be provided in part, provided that the non-disclosure of these data and respect for confidentiality can be guaranteed.
